# Severe bradycardia induced by postoperative nausea and vomiting: A case report

**DOI:** 10.1097/MD.0000000000034736

**Published:** 2023-08-25

**Authors:** Xiaoyu Li, Bo Lu

**Affiliations:** a Department of Anesthesiology, Ningbo NO. 2 Hospital, Ningbo, China.

**Keywords:** bradycardia, case report, PONV, vagal reflexes

## Abstract

**Introduction:**

Postoperative nausea and vomiting is a common complication for patients after anesthesia and surgery, which may result in increased parasympathetic activity, such as diaphoresis, pallor, or bradycardia. However, few cases of fatal bradycardia induced by postoperative nausea and vomiting have been reported before. Clinicians generally attribute bradycardia to certain anesthetics, instead of postoperative nausea or vomiting.

**Patient concerns:**

A fifty-year-old female with a history of well-controlled hypertension underwent elective radical mastectomy. When recovering from anesthesia in the post-anesthesia care unit, the patient experienced severe bradycardia accompanied by hypotension and unconsciousness, shortly after nausea and vomiting.

**Interventions:**

The patient received cardiopulmonary resuscitation immediately.

**Outcomes:**

Five minutes later, She recovered sinus rhythm and her vital sings tended to be stable. Three hours later, blood tests showed the N-terminal pro-B-type natriuretic peptide 127 pg/mL and cardiac troponin I 0.44 ng/mL, which peaked to 2.65 ng/mL 10 hours after the emergency. Electrocardiography revealed sinus rhythm, ST-segment depression in the inferior and anterior lateral leads, QTc prolongation, and left ventricular high voltage. Her serum cTnI continued to decline to 0.27 ng/mL on the 3rd day after surgery. She was discharged from the hospital on the fifth day and had no sequelae.

**Conclusion:**

Although postoperative nausea and vomiting occurs frequently, it should be kept in mind as a potential cause to blame for severe bradycardia or even life-threatening situations.

## 1. Introduction

Postoperative nausea and vomiting (PONV) is a common but unpleasant experience for patients after anesthesia and surgery, which may result in increased parasympathetic activity including diaphoresis, pallor, or bradycardia. However, very few cases of severe or near-fatal bradycardia induced by PONV have been published before. We herein describe a case of a patient who suffered hemodynamically unstable bradycardia induced by PONV in the Post-Anesthesia Care Unit (PACU). PONV-induced symptomatic bradycardia could be quite dangerous when the patient has a high vagal tone or with underlying cardiovascular conditions. We report this case to alert anesthesiologists that PONV could be a potential cause to blame for severe bradycardia or even life-threatening situations.

## 2. Case report

A 50-year-old woman, ASA II, presented for elective mastectomy due to breast tumor. She had mild hypertension which was well controlled by taking amlodipine and bisoprolol orally. She also had chronic active hepatitis B with entecavir tablets for therapy. The laboratory results and physical examination were all unremarkable. Electrocardiography (ECG) showed sinus rhythm and left ventricular high voltage. She didn’t take the echocardiography exam for she never felt any discomfort in her heart. She had no history of anesthesia before. Her BMI was 28 kg/m^2^ and she was a nonsmoker. Written informed consent for this report was obtained from the patient.

In the operating room, routine monitoring with noninvasive blood pressure, ECG, and pulse oximetry (SpO_2_) was applied which showed the heart rate (HR) of 60 to 80 beats/min, blood pressure (BP) of 120/80 mm Hg, SpO_2_ of 97% on room air. An i.v. line was inserted and infusion with Ringer’s lactate 500 mL was initiated. A continuous infusion of dexmedetomidine 50 µg was administered in 10 minutes. Followingly, anesthesia was induced with i.v. midazolam 2 mg, fentanyl 0.1 mg, cis-atracurium 3 mg, propofol 50 mg, and then the laryngeal mask was placed in proper condition. Remifentanil and sevoflurane were maintained during the operation. The operation was about 3 hours and the course of anesthesia/operation was uneventful.

In the PACU, the patient was extubated when her spontaneous breathing and consciousness recovered. A few minutes later, she complained of nausea and vomited about 10 mL of yellow bile-like liquid 10 minutes later. Suddenly, the ECG showed that HR dropped to 42 beats/min, and the sinus rhythm turned to ventricular escape rhythm. Simultaneously, SpO_2_ drops to 85%, and the BP was 65/36 mm Hg. The patient looked pale and her lips turned to be cyanosis. She was subsequently indifferent and could not respond to any instruction, with HR 40 beats/min, BP 50/30 mm Hg, and SpO_2_ 75%. Immediately, the anesthetist performed cardiopulmonary resuscitation. The patient was ventilated with mask and intubated simultaneously, with adrenaline 1 mg iv. Administered. The monitor showed partial pressure of end-tidal carbon dioxide (ETCO2) 4 cmH_2_O. About 5 minutes later, the patient recovered to sinus rhythm, with the heartbeat rising to 125 beats/min, BP 85/47 mm Hg, SpO_2_ 98%, EtCO_2_ 32 cmH_2_O. Pupils reflexes were tested normal. Artery blood gas showed acidosis with pH 7.08, PaCO_2_ 85 mm Hg, PaO_2_ 333 mm Hg, Hct 34%, HCO3^−^ 23.6 mmol/L, Lac 0.4 mmol/L, and electrolytes were normal (K^+^ 4.33 mmol/L, Na^+^ 149 mmol/L, Cl^-^ 107 mmol/L). The patient was transferred to the ICU when her vital signs were stable. Three hours later, blood tests showed the NT-proBNP 127 pg/mL and cardiac troponin I (cTnI) 0.44 ng/mL, which peaked to 2.65 ng/mL 10h after the emergency (Table 1). The ECG showed sinus rhythm, ST-segment depression in the inferior and anterior lateral leads, QTc prolongation and left ventricular high voltage. On the next day, the patient was extubated and transferred to the surgery ward. The cTnI continued to decline to 1.54 ng/mL, and then to 0.27 ng/mL on the third day after surgery. Seven days later, we did not find ST-segment depression in the inferior and anterior lateral leads and QTc prolongation in ECG. Then the patient was discharged in good general condition.

**Table 1:** Laboratory results of the patient.

	Time from severe bradycardia			
	3 h	10 h	20 h	2 d
cTnI (ng/mL)	0.44	2.65	1.54	0.27
CK-MB (ng/mL)	–	–	24	13
NT-proBNP (pg/mL)	127	–	–	–

cTnI = cardiac troponin I, CK-MB = creatinine kanase-myocardial band, NT-proBNP = N-terminal pro-B-type natriuretic peptide, “–” = means not applied.

The patient was hospitalized for 10 days after the operation when the suction axillary drainage was removed. During this post-op period, the patient did not either complain of any chest tightness, nor present nausea and vomiting. The ECG on 7th post-op day was unremarkable, which was almost the same as that before surgery.

## 3. Discussion

Postoperative nausea and vomiting are among the most common symptoms observed in PACU following general anesthesia or a surgical procedure. Typically, the incidence of PONV ranges between 25% and 30%.^[1]^ In rare cases, PONV can be associated with serious complications like suture dehiscence, esophageal rupture, or aspiration of gastric contents.^[2,3]^ But no severe bradycardia related to PONV was reported in the literature. The present report might be the first report that PONV caused severe bradycardia after anesthesia.

In most cases during the perioperative period, bradycardia is often caused by a sudden increase in the activity of the vagus nerve. It is generally known that vagal reflexes can induce sinus bradycardia, such as pressing the eyeball or carotid sinus, stimulating the pharynx, breath-holding, swallowing, severe coughing or doing Valsalva maneuver.^[4]^ Although sinus bradycardia is mostly asymptomatic, it can be a serious problem if the heart doesn’t pump enough oxygen-rich blood to the body. In this case, the patient experienced bradycardia accompanied by ventricular escape rhythm immediately after vomiting and nausea in PACU. Considering the sequences of these events, we deemed the cardiac arrhythmia to be triggered by nausea and vomiting, as the patient had no identifiable underlying cardiovascular conditions. Also, nausea and vomiting as manifestations of cardiovascular adverse events could be excluded. Firstly, the patient had no previous complaints of chest discomfort and she did not feel chest distress or pain after waking up in the ICU. Secondly, the peak of troponin in this patient was 10 hours after the onset of nausea and vomiting, which decreased and tended almost to normal 48 hours later. The troponin trend is clearly different from that of myocardial infarction in which situation the cTnI could last for at least 5 to 7 days. Therefore, we speculated the cTnI increase for this patient be caused by chest compressions and adrenaline, and the severe bradycardia after vomiting is probably due to reflex vagal excitability and activation of the baroreceptor.

It is well known that Valsalva maneuver (VM)-like activities, which increased intra-abdominal pressure and intrathoracic pressure, could be associated with vagal-mediated cardiovascular reflex initiated by such alterations.^[5,6]^ Nausea and vomiting are not rare causes of this kind of increase in parasympathetic tone, as we’ve often observed in the clinic that vagal nerve stimulation could be produced by various gastrointestinal problems (especially nausea or vomiting).^[7]^ For this case, it is noted that the patient complained of tightness of the chest strap. At this point, nausea and vomiting lead to an increase in intra-abdominal pressure, and then caused rapid rise in intra-thoracic pressure because she was unable to expand her chest with a tight strap bound up. Consequently, the increase in the thoracic pressure triggered the carotid sinus baroreceptor reflex, which therefore, simultaneously activates the vagal outflow to the heart and inhibits sympathetic activity to the vasculature. As a result, an abrupt drop in blood pressure and a sudden reduction in heart rate. The reflex, though universal, actually varies from person to person according to the level of reactivity to triggers and excitability of the vagal tone.^[8]^

The limitation of this case was that we did not save ECG images when the event happened. In addition, the treatment was not quite appropriate. In this case, the pulse should be assessed as the first step, and it would have been better to try intravenous atropine and fluids, other than ardiopulmonary resuscitation.

## 4. Conclusion

PONV-induced symptomatic bradycardia is rarely known but actually occurs. It could be quite dangerous when the patient has a high excitability of the vagal tone or with underlying cardiovascular conditions. Although PONV occurs frequently in the perioperative period, it should be kept in mind as a potential cause to blame for severe bradycardia and even life-threatening situations. However, in the literature database, we have found no evidence that the increase in gastric pressure caused by nausea and vomiting can lead to severe bradycardia, and the mechanism of nausea and vomiting-induced arrhythmia needs further research.

## Author contributions

**Resources:** Xiaoyu Li.

**Supervision:** Bo Lu.

**Writing – original draft:** Xiaoyu Li.

**Writing – review & editing:** Bo Lu.
